# The comparative science of “self-control”: what are we talking about?

**DOI:** 10.3389/fpsyg.2015.00051

**Published:** 2015-01-30

**Authors:** Michael J. Beran

**Affiliations:** Language Research Center, Georgia State UniversityAtlanta, GA, USA

**Keywords:** self-control, delay of gratification, comparative cognitive science, behavioral inhibition, intertemporal choice

There has been great interest in comparative cognitive science in a suite of behaviors that have come to be subsumed under the term “self-control.” Historically in comparative psychology self-control was defined behaviorally as the choice of a larger or better but more delayed reinforcer over a smaller or less preferred but less delayed reinforcer (Rachlin and Green, 1972; Ainslie, 1974; Grosch and Neuringer, 1981; Logue, 1988). More recently, however, self-control has been discussed more in terms of being a capacity or ability rather than a behavioral pattern, even to the extent of some researchers suggesting that self-control may be a limited capacity resource that can be depleted, strengthened, or improved with practice (e.g., Baumeister et al., 2007). A terminological morass has emerged in this area, with varying definitions of this term, as well as other perhaps related terms such as patience, willpower, delay of gratification, and intertemporal choice. The goal here is not to untangle that mess of terminology, but to comment on two recent papers in comparative psychology that attempted to provide insights into the “evolution of self-control,” with varying degrees of success. I also will provide the beginnings of a framework for a terminology for assessing these behavioral forms of self-regulation and inhibition.

In one new paper, Stevens (2014) discussed the role that ecologically valid tests play in understanding the evolution of self-control (also Stevens et al., 2005a,b). These tests are excellent ways of looking at how animals value time, distance, and outcome options, and they remind us that ultimately the behavior we see in laboratory tests should relate to the pressures a given species might face in the natural environment. Stevens (2014) also addressed three competing hypotheses that might predict basic waiting times in an intertemporal choice task in which there is a choice between a smaller, sooner reward and a larger, later reward. Presumably, waiting longer is evidence of self-control because one obtains more reward, but at the cost of tolerating the delay. The *body size hypothesis* predicts that larger species should wait longer than smaller species because of allometric relationships that describe how morphological, physiological and behavioral measures scale with body size (Schmidt-Nielsen, 1984). According to this hypothesis (sometimes also called the *metabolic hypothesis*; Tobin and Logue, 1994), smaller animals have faster metabolic rates, and thus should tend to choose shorter delay periods to any food relative to animals with larger bodies (see Speakman, 2005). Lifespan also scales with body size, and thus longer living animals should have longer waiting times than shorter living animals according to this hypothesis. The *cognitive ability hypothesis* predicts that longer wait times would relate to generally higher levels of cognition across species. This hypothesis directly links to the emerging findings in human psychology that good delay of gratification and high self-control are predictive of more positive life outcome measures (e.g., higher education achievements) that also relate to general intelligence (e.g., Duckworth and Seligman, 2005; Shamosh et al., 2008). As a proxy for general cognitive abilities across primate species, Stevens (2014) used relative and absolute brain size, measures that have shown clear relations with other aspects of primate cognition (e.g., Gibson et al., 2001; Deaner et al., 2007). Finally, the *social brain hypothesis* predicts that species that live in more complex groups of many individuals should wait longer than those that live in less complex groups. This is because these individuals need to more often employ inhibitory strategies as they monitor and engage in social events occurring around them such as tracking the fission and fusion events within the group (Dunbar, 2009), and some previous research seems to support this hypothesis (Amici et al., 2008).

To assess these hypotheses in terms of their ability to predict intertemporal choices, Stevens (2014) calculated mean values for indifference points between smaller-sooner (SS) and larger-later (LL) rewards as the measure of self-control. He also used data for each species for body mass, measures of brain volume, home range size, lifespan, and group size. What he found was that the allometric variables predicted the ability to wait for delayed rewards across 13 species of primates. Specifically, he found that a composite allometric factor that included body mass, absolute brain size, lifespan and home range size predicted waiting times. Relative brain size and social group size did not, and thus he concluded that certain selective pressures have acted to shape intertemporal choices made by animals. He noted that one limitation of his study involved the methods used to measure intertemporal choice. Although those methods were fairly consistently applied across the studies he analyzed, these intertemporal choice tasks may not specifically have assessed self-control vs. other decision mechanisms.

Using a different approach, MacLean et al. (2014) assessed 567 animals representing 36 species on two tasks. In the A-not-B task, subjects first learned that food was always in one location from a choice array, but then it was moved in the last trial while the animal watched the item move. If animals searched in the usual location instead of switching to the new location this was considered an error, and it suggested that the animals could not control their response to the old location in order to reach toward the new one. In the cylinder task, subjects first found a piece of food hidden inside an opaque cylinder and had to reach around the side to retrieve it. Then, a transparent cylinder was shown instead of the opaque cylinder. Subjects still had to reach around the cylinder to grab the food rather than reaching for the food as they saw it through the transparent cylinder. Direct reaches that were blocked by the cylinder were considered errors. Absolute brain volume best predicted performances of these species in successfully obtaining the rewards. Social group size was not a strong predictor of species differences. MacLean et al. (2014) suggested that these results implicated evolutionary relationships between dietary breadth, absolute brain volume, and self-control. Although I applaud the effort to provide such a comprehensive comparative database, this conclusion does not provide insights to the evolution of self-control. Rather, MacLean et al. (2014) assessed one form of behavioral inhibition (a construct which itself can be viewed as multifaceted), and not self-control, at least as it is traditionally defined, and as I suggest it should be defined.

I do not have space here for an exhaustive framework to incorporate the behavioral data that are being generated and discussed in papers such as MacLean et al. (2014) and Stevens (2014). Instead, I want to focus on generating a framework for what should (and what should not) be called self-control. I define self-control as the ability or capacity to obtain an objectively more valuable outcome rather than an objectively less valuable outcome though tolerating a longer delay or a greater effort requirement in obtaining that more valuable outcome. A self-control task has three essential features. First, there must be at least two known options available, and each must be available to the subject through differential responses. Second, those options each must be valuable to some extent but must be differentially preferred by the subject, *if all else is the same*. Third, there must be a cost (generally in terms of time delay, but also perhaps in terms of effort exerted) for obtaining the more preferred outcome. Self-control tasks are thus tasks that require decisions, not just inhibition of prepotent responses.

By this definition, self-control and behavioral inhibition are not synonymous. Rather, self-control such as that assessed in inter-temporal choice tasks or delay of gratification tasks is but one form of behavioral inhibition, although I would argue it is one of the most advanced and most challenging forms for many species and many individuals within a species. The critical point is that a task requiring behavioral inhibition does not necessarily require self-control, and that this error in our use of terminology has had distracting effects on what we can say about self-control and its evolutionary emergence in nonhuman animals and humans. And, as pointed out by one of the reviewers of this article, some circumstances that require *increased activity* rather than inhibition to obtain the better outcome require self-control (for example, choosing to work longer for more pay rather than leaving work early).

Self-control also is not synonymous with delay of gratification. Delaying gratification is what one does in a subset of instances in which self-control is employed. Delayed gratification tasks such as the Marshmallow test (e.g., Mischel and Ebbesen, 1970) are self-control tasks because any task that requires a subject to do nothing but wait, toward the goal of getting a bigger or better outcome, meets the three features outlined above. There is the available reward (e.g., one marshmallow now) and the later reward for which one must wait (e.g., two marshmallows later). If there was no waiting period, the second option would be preferred in general because it is bigger or better (e.g., 2 vs. 1 marshmallow). And, there is a time cost imposed for obtaining that larger or better reward. Intertemporal choice tasks such as those in the Stevens (2014) paper also are self-control tasks, although one must be careful about how to interpret some intertemporal choice tasks that make use of pointing responses toward food rewards. Those testing approaches may lead to overestimates of self-control, as recently demonstrated in capuchin monkeys using the Hybrid Delay task (Paglieri et al., 2013). In that task, subjects first chose between an SS option and an LL option. If they chose the LL option, they had to wait for items to accumulate, one by one, within reach, and the accumulation ended when any items were eaten. This task assessed objective errors in which the LL option was selected but the accumulation was terminated before enough items were collected to match what the SS option had offered. Capuchin monkeys made many such errors although chimpanzees showed better self-control by often choosing the LL option but also waiting for it to accumulate fully (Beran et al., 2014). Other tasks that meet the criteria for assessing self-control include delayed exchange tasks (e.g., Dufour et al., 2007; Pele et al., 2010), accumulation tasks (Beran, 2002; Beran and Evans, 2006), some token collection or exchange tasks (Jackson and Hackenberg, 1996; Hackenberg and Vaidya, 2003; Judge and Essler, 2013), and similar approaches (e.g., Evans and Westergaard, 2006; Evans, 2007; Bramlett et al., 2012).

Critically, one must then recognize what are not self-control tasks. A-not-B tasks, inhibitory motor tasks, flanker tasks, Stroop tasks, Go No-Go tasks, and others of this general form do not fall under the umbrella of self-control assessment. They can be tasks that require degrees of behavioral inhibition, and thus are highly relevant to understanding aspects of cognitive control and self-regulation. But, not all forms of behavioral inhibition rely on self-control. For example, Maclean et al.'s (2014) cylinder task requires inhibiting reaching directly toward the food, but there is no second option for getting less food more quickly, and no decision between two temporal durations (or effort levels) and two outcomes, one of which might be better now, but the other of which is better in the long term. Thus, MacLean et al. (2014), despite their title, instead have assessed the evolution of certain forms of behavioral inhibition and motoric self-regulation in animals–not self-control. One should applaud the impressive efforts to provide a truly comparative approach to studying different forms of behavioral inhibition. But Stevens (2014) has provided clearer information on the evolution of self-control, and a stronger road map for future research on this issue.

Much work remains to be done to understand self-control capacities in nonhuman species. For example, there are concerns about exactly what different tasks involving intertemporal choice measure not only for nonhuman animals (e.g., Addessi et al., 2013) but also for humans (Duckworth and Kern, 2011). And, as Stevens (2014) noted, we need more cross-species assessments using some of the other methods that are designed to measure self-control such as delay of gratification tasks (e.g., Grosch and Neuringer, 1981; Beran et al., 1999), delayed exchange tasks (e.g., Dufour et al., 2007; Pele et al., 2010; Judge and Essler, 2013), accumulation tasks (Beran, 2002; Evans and Beran, 2007; Vick et al., 2010; Parrish et al., 2014), and other variations on these methods. With those data, one could compare the resulting correlations between performance and allometric factors to see if they match what Stevens found for the intertemporal choice task. This approach would provide a strong assessment of the evolution of self-control as it relates to the suite of tasks and circumstances in which self-control abilities would be necessary for maximizing outcomes. Hopefully, future discussions of the behaviors (and underlying capacities and mechanisms) of animals in situations that require different forms of inhibition will carefully consider what the appropriate level of interpretation should be, otherwise we run the risk of reducing all forms of inhibition to being the same as self-control.

## Conflict of interest statement

The author declares that the research was conducted in the absence of any commercial or financial relationships that could be construed as a potential conflict of interest.
